# New Advances in Palliative Care—State of the Field, Its Challenges and Advances at the End of the Year 2025

**DOI:** 10.3390/healthcare14020206

**Published:** 2026-01-14

**Authors:** Georg Bollig, Erika Zelko

**Affiliations:** 1 Department of Anesthesiology, Intensive Care, Palliative Medicine and Pain Therapy, Helios Klinikum Schleswig, St. Jürgener Str. 1-3, 24837 Schleswig, Germany; 2 Department of Palliative Medicine, Faculty of Medicine and University Hospital, University of Cologne, Kerpener Str. 62, 50937 Köln, Germany; 3 Last Aid Research Group International (LARGI), 24837 Schleswig, Germany; erika.zelko@jku.at; 4 Letzte Hilfe Deutschland gGmbH, 24837 Schleswig, Germany; 5 Institute for Palliative Medicine, Medical Faculty, University of Maribor, Taborska cesta 9, 2000 Maribor, Slovenia; 6 Institute for General Medicine, Medical Faculty, Johannes Keppler University, Altenberger Str. 69, 4040 Linz, Austria

## 1. Introduction

In recent decades, palliative care has become an essential component of modern healthcare systems. According to the World Health Organization (WHO), “Palliative care is an approach that improves the quality of life of patients and their families facing problems associated with life-threatening illness, through the prevention and relief of suffering by means of early identification and impeccable assessment and treatment of pain and other problems, physical, psychosocial and spiritual” [1]. Despite its recognized importance, an estimated 56.8 million people worldwide require palliative care each year, while only approximately 14% of those in need actually receive it [1]. This underlines the urgent need to strengthen and expand palliative care services around the world. Today we know that the need for palliative care is high, not only in cancer patients but also in patients with chronic non-oncologic conditions [2]. Unfortunately, palliative care is still frequently associated solely with imminent death or limited to oncology patients. Awareness remains insufficient among both healthcare professionals and the general public that individuals with other chronic, life-limiting conditions—as well as people from diverse ethnic, cultural, and social backgrounds—may also significantly benefit from palliative care delivered by community health services and specialized palliative care teams. To address these gaps, new approaches that promote inclusivity and foster collaboration among communities, informal carers, and healthcare professionals are essential. The Lancet Commission on the Value of Death and a number of other experts have stated that there is a need to improve death literacy, knowledge, and skills in palliative care and end-of-life care for everyone [3,4,5,6]. The COVID-19 pandemic further highlighted vulnerabilities in palliative care provision, as established services were disrupted and innovative methods of care delivery had to be developed. Digital solutions are often not fully implemented in palliative care and there is a need for development of digital infrastructures in the field of palliative care [7]. Telehealth and telepalliative care emerged as important tools to maintain communication and support patients and families during this unprecedented period [8,9,10]. Other new ways of including technology in palliative care provision are in the pilot phase, as, for example, the drone-based delivery of medication [11]. By the end of 2025, palliative care has continued to evolve globally. When Cicely Saunders started the palliative care and hospice movement in the 1960s, the first advances were the recognition of the needs of seriously ill and dying people by healthcare professionals, establishing hospices as places for both short-term and long-term palliative care, end-of-life care for those in need and of course the efforts to provide symptom relief for distressing symptoms, first of all providing access to opioids as morphine to treat pain and dyspnea. Later on, the focus broadened to a number of diseases other than cancer and to an increased availability of palliative care in the communities. Palliative Care and Palliative Medicine became in the course of this recognized fields for nurses, physicians and other healthcare professionals. Today, palliative medicine is recognized as a distinct medical specialty in some countries as Great Britain and Australia, while in others like Germany and the USA remains a subspecialty. In 2025, Dr. Balfour Mount—widely regarded as the father of palliative care in Canada and the physician who coined the term “palliative care”—passed away [12], marking a significant moment in the history of the field. His death reminds us to look back and acknowledge the achievements already made in palliative care but also to focus on the challenges that lie ahead. Looking back at the relatively short history of palliative care, the most important recent developments in the field from our point of view have been the early integration of palliative care in oncology, the expansion of the role of palliative care for patients with other chronic life-limiting diseases than cancer and telepalliative care in order to reach more people with specialized palliative care in rural areas or despite other hindrances to personal treatment at home or in an outpatient clinic. The most important gaps that should be addressed in the future include the knowledge about palliative care provision for all in need, taking into account the increasing need for palliative care and the demographic change, the broad implementation of early palliative care, how to improve public awareness and inclusion of informal carers in palliative care provision at home, the cooperation of professionals with lay people in compassionate communities and how to include new technologies as telepalliative care in the best interest of patients, relatives and healthcare professionals.

## 2. An Overview of Published Articles in This Special Issue

The aim of this Special Issue, “New Advances in Palliative Care”, is to highlight recent developments and ongoing challenges related to the inclusion of diverse patient groups, palliative care during pandemics, telepalliative care, and the integration of modern technologies into palliative practice. The articles included in this Special Issue address a broad spectrum of topics within this framework and reflect perspectives from patients, relatives, volunteers, and healthcare professionals. Grimm et al. (contribution 1) present an approach to improve end-of-life outcomes through a minimal invasive intervention (MINI), which shows potential for enhancing patient-centered care and palliative care delivery in acute hospital settings (contribution 2). In the context of primary palliative care in the community, the role of primary healthcare professionals is crucial for identifying patient needs and ensuring timely referral to specialist services when required (contribution 3). Düzgün et al. highlight the potential contribution of university students as volunteers within caring communities (contribution 4), emphasizing the value of civic engagement in end-of-life care. Informal caregivers play a vital role in providing palliative and end-of-life care at home and should be adequately supported by specialized palliative care teams and through education for the public (contribution 5, contribution 12). Telehealth can serve as an additional modality to support informal caregivers in the home setting (contribution 5). However, caregivers’ needs are often insufficiently addressed, leaving them vulnerable to emotional strain and financial burden (contribution 6). Hagen and Zelko demonstrate that the likelihood of dying at home is strongly associated with the availability of home-based healthcare services. Their registry-based study showed that patients with breast cancer and heart failure were more likely to die at home than patients with dementia (contribution 7). This finding underscores the urgent need for broad implementation of caring communities and equitable access to palliative care irrespective of diagnosis. MacAden and Muirhead introduce the Dementia Education for Workforce Excellence (DEWE) program, an innovative educational resource that promotes person- and relationship-centered dementia care across all stages of the disease trajectory (contribution 8). Furthermore, a systematic review by Farrukh et al. suggests that re-irradiation may prolong survival in patients with progressive diffuse intrinsic pontine glioma as part of palliative treatment strategies (contribution 9). Public awareness remains a cornerstone of effective palliative care delivery. Spary-Kainz et al. demonstrate that public information campaigns can positively influence the home care of seriously ill individuals (contribution 10). Herrera-Gomez et al. describe the challenges connected to identifying palliative care needs and the referral to specialized palliative care (contribution 11). Bollig et al. report on ten years of practical and scientific experience with Last Aid Courses as means for Public Palliative Care Education. Educational measures like the LAC can contribute to raise public awareness for the topics of death, dying, and grief and may encourage lay people to participate in end-of-life care provision in the community.

## 3. Conclusions

Collectively, the contributions in this Special Issue enrich the growing body of palliative care knowledge grounded in practice, theory, and research from diverse global contexts. They show that the cooperation between healthcare professionals, patients, relatives, and lay people in a compassionate community approach can be a solution to provide palliative care for more patients in need. Together, we have to strive to make palliative care available for more people in need. As part of this collaboration and joint effort to improve palliative care provision around the world, further research and sustained implementation efforts are required to translate the scientific evidence into equitable, worldwide palliative care provision. With the contributions in this issue, some knowledge gaps could be addressed, as described above. Nevertheless, the field of palliative care practice and research has to be developed further. Future directions for improvement in practice and research are as follows:

○ Early integration of Palliative Care;
○ Make Palliative Care available everywhere for all in need;
○ Public awareness and participation of lay persons in Palliative Care provision in compassionate communities;
○ New technologies to support Palliative Care (e.g., telepalliative care);
○ Encourage an open public debate on assisted dying.

Although we know that early integration of palliative care in the course of different diseases is meaningful, early integration is still lacking in many clinical situations. Up to 10% of patient visits in the Emergency Department are due to palliative care needs [13]. Early recognition of these needs can help to reduce symptom burden. More efforts are needed to make early integration of palliative care a standard throughout healthcare, including all emergency departments. As shown above, the overall need for palliative care worldwide is much higher than the actual numbers of palliative care provision [1]. Therefore, we need a combination of different measures in order to be able to address the future palliative care needs worldwide. Based on the findings presented in this Special Issue and our broader perspective, future challenges in palliative care include ensuring access for all individuals in need and developing innovative models to support informal caregivers both financially and through practical and psychological assistance. As articulated by Kellehear, palliative and end-of-life care are “everybody’s business” [14]. His “95% rule” emphasizes that seriously ill and dying individuals spend only about 5% of their final year of life in direct contact with healthcare services, while the remaining 95% depends on informal caregivers—family members, friends, and community members—to provide care [14]. Public awareness and contribution of lay people is urgently needed. One measure to improve public knowledge is public palliative care education throughout society starting already in school. The example of Last Aid Courses for the public is shown in contribution 12. These LACs can improve palliative care knowledge and encourage people to participate in end-of-life provision in the model of compassionate communities (contribution 12) [14]. The integration of digitalization and new technologies as telepalliative care and other technical innovations may help to provide palliative care for more people living in rural areas without direct access to specialist palliative care [7,8,9,10,11]. Another important challenge for the future is an open public debate about assisted suicide and the connection to palliative care. In many countries, a number of people are for or against it. This is also true for palliative care practitioners [15]. Therefore, an open debate of all people, including healthcare professionals, ethicists, and politicians, is needed in order to find practical ways to support people at the end of life. The availability of palliative care for everyone in need, adequate funding of palliative care services [16], and the best possible symptom relief for suffering patients are from our point of view the most important measures to address the wish for assisted death.

To meet the increasing demand for palliative care within communities, enhanced collaboration between lay people and healthcare professionals, supported by a caring community approach, is essential. Telepalliative care holds significant promise, but further research and practice-based models are needed to integrate this approach effectively into routine care and community structures. When everyone contributes, we can collectively strive to ensure compassionate care for all those in need.
